# Effect of Dental Local Anesthetics on Reactive Oxygen Species: An In Vitro Study

**DOI:** 10.7759/cureus.63479

**Published:** 2024-06-29

**Authors:** Hidetaka Kuroda, Shota Tsukimoto, Azuma Kosai, Noriko Komatsu, Takehito Ouchi, Maki Kimura, Aiji Sato-Boku, Ayaka Yoshida, Fumihiko Yoshino, Takahiro Abe, Yoshiyuki Shibukawa, Takuro Sanuki

**Affiliations:** 1 Department of Dental Anesthesiology, Kanagawa Dental University, Kanagawa, JPN; 2 Department of Oral and Maxillofacial Surgery, Kanagawa Dental University, Kanagawa, JPN; 3 Department of Physiology, Tokyo Dental College, Tokyo, JPN; 4 Department of Anesthesiology, Aichi Gakuin University, Nagoya, JPN; 5 Department of Liberal Arts Education, Kanagawa Dental University, Kanagawa, JPN; 6 Department of Pharmacology, Kanagawa Dental University, Kanagawa, JPN

**Keywords:** superoxide anion, reactive oxygen species, prilocaine, oxidative stress, lidocaine, hydroxyl radical, articaine, antioxidative effects

## Abstract

Introduction

Oxidative stress, an imbalance between reactive oxygen species (ROS) production and antioxidant defenses, plays an important role in various dental diseases. Local anesthetics are frequently used in dentistry. The potential antioxidant activity of dental local anesthetics can contribute to dental practice. Therefore, this study aimed to investigate the ROS-scavenging activities of three commonly used dental local anesthetics, lidocaine, prilocaine, and articaine, focusing on their effects on hydroxyl radicals (HO^•^) and superoxide anions (O_2_^•−^).

Materials and methods

The electron spin resonance (ESR) spin-trapping technique was employed to specifically measure the ROS-scavenging activities of these local anesthetics at varying concentrations.

Results

Lidocaine, prilocaine, and articaine exhibited concentration-dependent HO^•^-scavenging activities, with IC_50_ values of 0.029%, 0.019%, and 0.014%, respectively. Lidocaine and prilocaine showed concentration-dependent O_2_^•−^-scavenging activity, with IC_50_ values of 0.033% and 0.057%, respectively. However, articaine did not scavenge O_2_^•−^.

Conclusions

The proactive use of dental local anesthetics may mitigate oxidative injury and inflammatory damage through direct ROS scavenging. However, further research is needed to elucidate the specific mechanisms underlying the antioxidant effects of these dental local anesthetics and their potential impact on the dental diseases associated with oxidative stress.

## Introduction

Oxidative stress occurs when there is an imbalance between the production of reactive oxygen species (ROS) and the ability of the body to neutralize them through antioxidant defenses. Oxidative stress plays a significant role in various aspects of medicine as it is involved in the pathogenesis of various diseases and the aging process [1,2]. Notably, oxidative stress has been implicated in the pathogenesis of diseases, such as cardiovascular and neurodegenerative diseases, metabolic disorders, chronic inflammatory diseases, cancers, aging, and age-related diseases [1-3]. Oxidative stress also influences several pathologies in dentistry and oral maxillofacial surgery. Increased oxidative stress markers have been observed in patients with periodontitis, suggesting a link between ROS and rapid destruction of periodontal tissue [4]. ROS damage the DNA and promote cell proliferation and the development of oral squamous cell carcinoma and other types of oral cancers [5,6]. Therefore, oxidative stress may play a role in medication-related jaw osteonecrosis by inhibiting bone healing and promoting inflammation [7,8]. Additionally, oxidative stress is involved in dental caries, oral lichen planus, oral submucous fibrosis, and aphthous stomatitis [9-12].

ROS are a group of independently existing molecules that possess at least one oxygen atom and one or more unpaired electrons. ROS includes various oxygen-containing free radicals, such as hydroxyl radical (HO^•^), superoxide anion (O_2_^•−^), hydrogen peroxide, peroxyl radicals, hydroperoxides, and alkoxyl radicals [13]. HO^•^ is one of the most powerful oxidizing agents among free radicals and can cause significant cellular damage by inducing lipid peroxidation, protein oxidation, and DNA strand break [14-16]. O_2_^•−^ are primarily generated as byproducts of cellular respiration in the mitochondrial electron transport chain or produced by other enzymes, such as NADPH oxidases and xanthine oxidase [17]. Importantly, high levels of O_2_^•−^ can lead to lipid peroxidation, protein oxidation, and DNA damage [18].

Local anesthetics are among the most frequently used drugs in dentistry. Local anesthetics are broadly classified as amide- or ester-based and are used for pain relief during dental practice. However, only certain amide-based local anesthetics are injected locally during dental practice [19]. While oxidative stress is associated with dental diseases, research on the ROS-scavenging activity of local anesthetics used in dental practice has largely focused on lidocaine, with limited studies on other local anesthetics. Therefore, we hypothesize that dental local anesthetics other than lidocaine may also scavenge ROS. This study aimed to investigate the ROS-scavenging activities of three commonly used dental local anesthetics (lidocaine, prilocaine, and articaine), specifically examining their ability to inhibit HO^•^ and O_2_^•-^. 

## Materials and methods

Evaluation of ROS‐scavenging activities using the ESR spin-trapping technique

**Figure 1:**
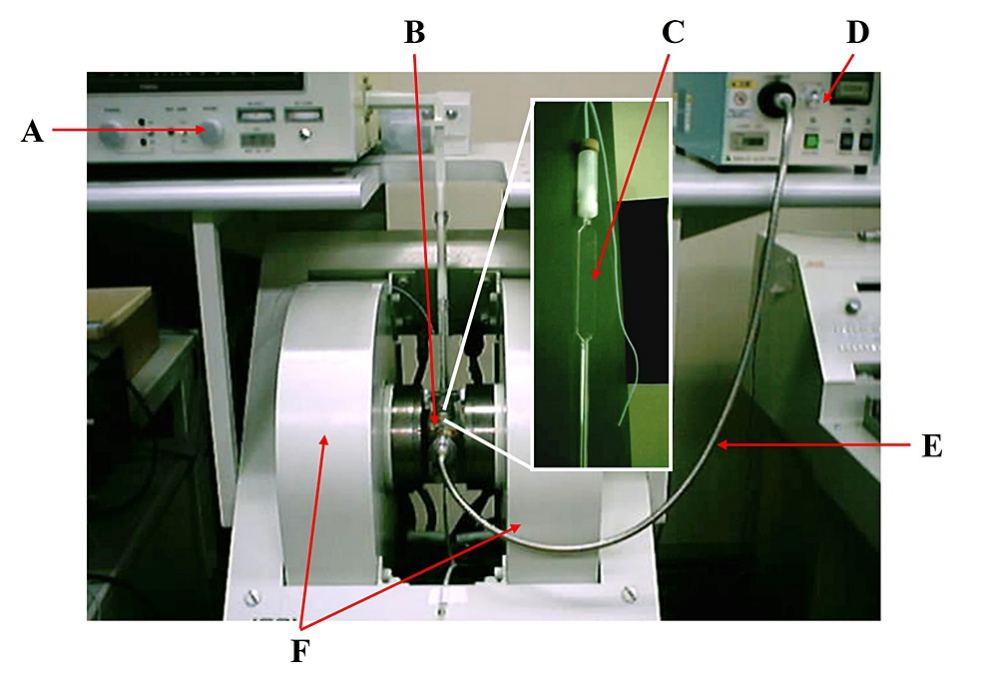
ESR instrument (A) Microwave generator and bridge, (B) ESR resonator cavity, (C) ESR quarts flat cell, (D) UV curing system (UVF-204S), (E) optical fiber, and (F) magnet. ESR, electron spin resonance; UV, ultraviolet

To test our hypothesis, we measured the ROS scavenging activities of the local anesthetics via the electron spin resonance (ESR) spin-trapping technique, using the X‐band spectrometer (JES‐RE 1X, JEOL, Tokyo, Japan) (Figure 1) [20-22]. The spectrometer was used under the following settings: a microwave power of 8.00 mW, magnetic field of 335.8±7.5 mT, field modulation width of 0.079 mT, sweep time of 1 min, and time constant of 0.03 s. Data acquisition was achieved using WIN RAD ESR Data Analyzer software ver.1.30 (JEOL). The gain was set to 320, and the software recorded 4096 data points. HO^•^ and O_2_^•−^ were generated a minimum of six times.

Solutions and reagents

HO^•^ was generated using 100 mM hydrogen peroxide (H_2_O_2_, FUJIFILM Wako Pure Chemical Industries, Osaka, Japan) under ultraviolet (UV) irradiation. UV light was generated using a UV curing system (UVF-204S, SAN-EI ELECTRIC, Osaka, Japan) with the following settings: emission wavelength, 365 nm; power, 100 mW; duration, 10 s (Figure 1) [20,21]. O_2_^•−^ was generated using 3.0% H_2_O_2_ and 0.3 wt% anatase-formed titanium oxide (TiO_2_, FUJIFILM Wako Pure Chemical Industries) as photocatalysis under UV irradiation at the following setting: emission wavelength, 365 nm; power, 100 mW/cm^2^; duration, 1 min [21,23]. For the ESR spin-trapping agent, we used 1 mM and 5 mM CYPMPO (5-(2,2-dimethyl-1,3-propoxycyclophosphoryl)-5-methyl-1-pyrroline-N-oxide) (Radical Research, Tokyo, Japan) for HO^•^ and O_2_^•−^, respectively [20,21,23].

The intensity of the CYPMPO-OH^•^ and -O_2_^•−^ spin adduct was measured at varying concentrations of local anesthetics as follows: lidocaine hydrochloride monohydrate (2-diethylamino-N-(2,6-dimethylphenyl)acetamide hydrochloride monohydrate) (lidocaine, Sigma-Aldrich, MO, USA), prilocaine hydrochloride (N-(2-methylphenyl)-2-(propylamino)propanamide hydrochloride) (prilocaine, Sigma-Aldrich), and articaine hydrochloride (4-methyl-3-[[1-oxo-2-(propylamino)propyl]amino]-2-thiophenecarboxylic acid methyl ester hydrochloride) (articaine, Sigma-Aldrich). Notably, the local anesthetics were diluted with distilled water.

Statistical analysis

To establish a baseline, the spectral intensity after the addition of distilled water was designated as *I*_0_. The spectral intensity corresponding to each local anesthetic was labeled *I*. The relative spectral intensity (%Intensity of *I*/*I*_0_) was assessed by calculating the *I*:*I*_0_ ratio. The concentration dependence of the local anesthetic reaction was determined by fitting the data to the following equation: A=Amax/[1+(IC_50_/[X]_0_)^h^]+Amin, where IC_50_ is the half-maximal inhibitory concentration for each local anesthetic with a Hill coefficient (h) of 1. Amax and Amin are the maximal and minimal responses, respectively [24,25]. [X]_0_ indicates the concentration of local anesthetic applied.

Data are expressed as the mean ± standard deviation (SD) of n observations, where n represents the number of separate experiments. Statistical significance was determined using one-way ANOVA with Dunnett's multiple comparisons. Statistical significance was set at *p*<0.05. All statistical analyses were performed using GraphPad Prism 7.05 (Graph Pad Software, La Jolla, CA, USA).

## Results

HO^•^-scavenging activities of dental local anesthetics

**Figure 2:**
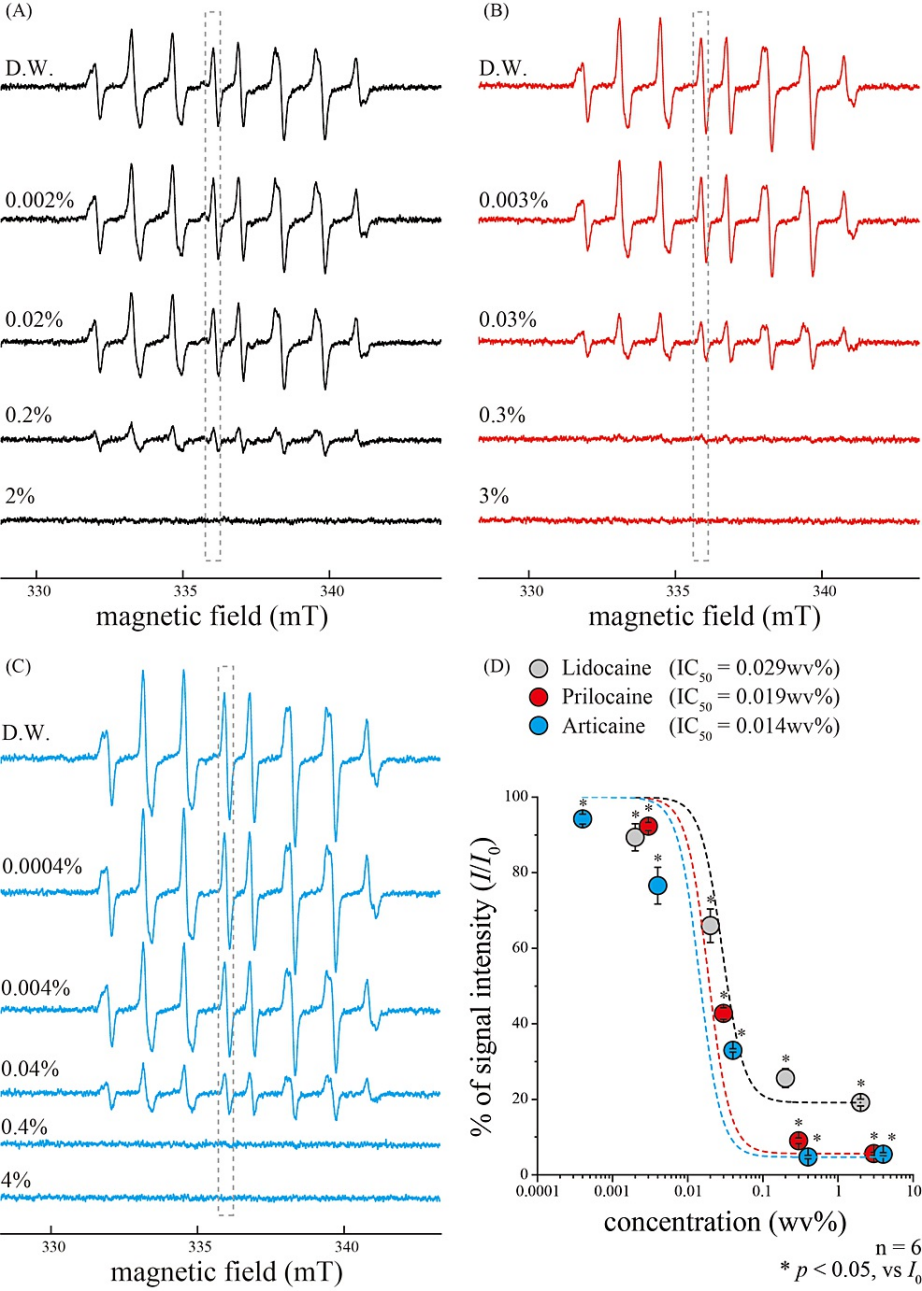
Dose-dependent relationships between hydroxyl radical signal intensities and dental local anesthetics (A-C) Representative typical ESR spectra of HO^•^ in response to various concentrations of lidocaine (A), prilocaine (B), and articaine (C). Signal intensities (in grey dotted boxes) were inhibited by each local anesthetic in a concentration-dependent manner. (D) Concentration-response relationships of different local anesthetics. Data points illustrate *I*/*I*_0_ as functions of each local anesthetic concentration. Grey circles represent lidocaine; red circles represent prilocaine; and blue circles represent articaine. Curves (solid lines) were fitted according to the equation described in the text. Each data point represents the mean ± SD of data from six separate experiments. Significant differences between data points are indicated using asterisks. *p<0.05. D.W., distilled water; IC50, the 50% inhibitory concentration; ESR, electron spin resonance; SD, standard deviation

To determine the antioxidant properties of dental local anesthetics, we examined the HO^•^-scavenging activities of lidocaine, prilocaine, and articaine. Notably, distilled water exhibited strong HO^•^ generation (Figure 2A-2C). However, HO^•^ generation was significantly inhibited by lidocaine (Figure 2A), prilocaine (Figure 2B), and articaine (Figure 2C) in a concentration-dependent manner, with IC_50_ values of 0.029, 0.019, and 0.014 w/v%, respectively (Figure 2D).

O_2_^•−^-scavenging activities of dental local anesthetics

**Figure 3:**
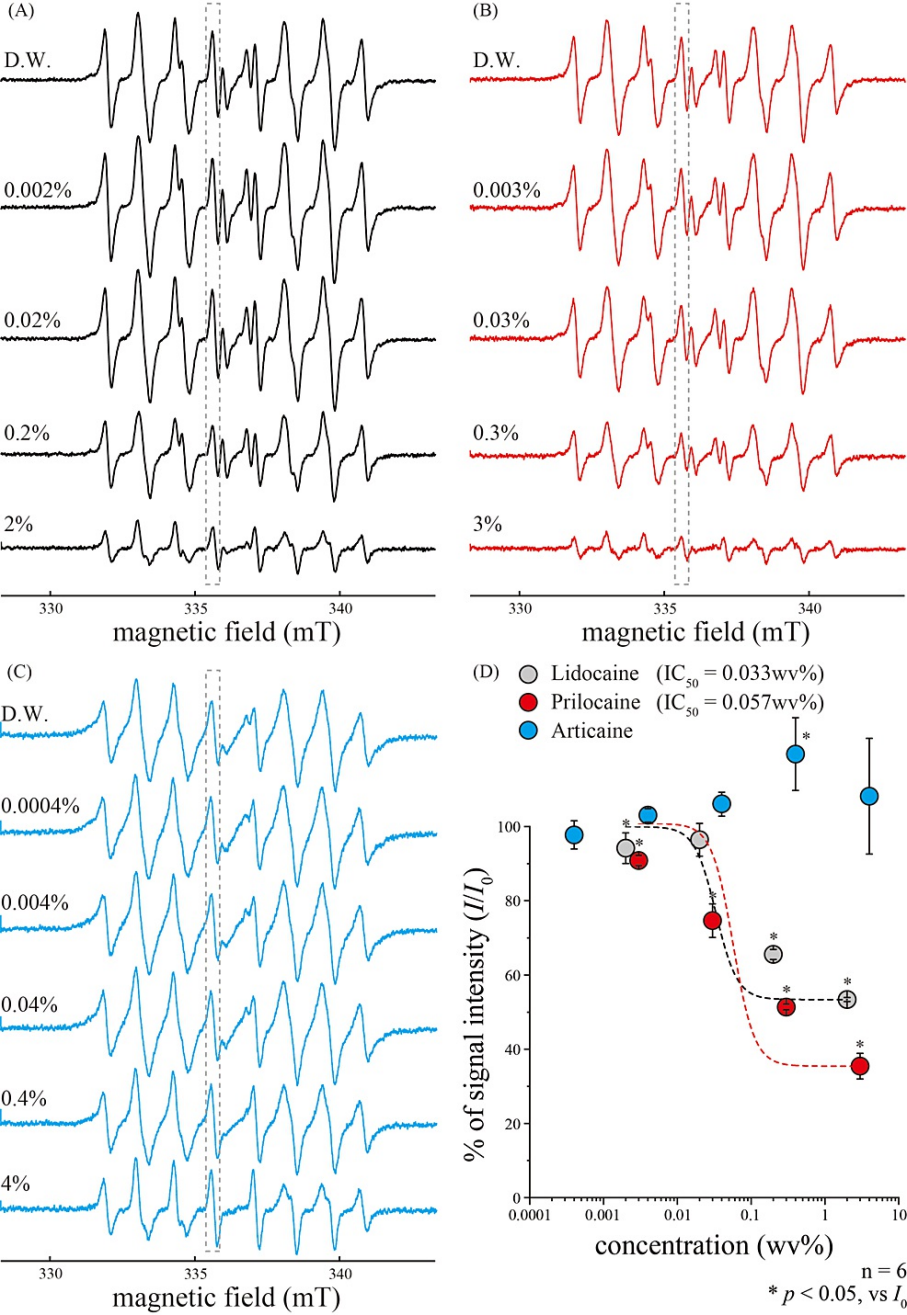
Dose-dependent relationships between superoxide anion signal intensities and dental local anesthetics (A-C) Representative typical ESR spectra of O_2_^•−^ in response to different concentrations of lidocaine (A), prilocaine (B), and articaine (C). Signal intensities (in grey dotted boxes) of D.W. were inhibited by lidocaine (A) and prilocaine (B) in a concentration-dependent manner. (D) Concentration-response relationships of different local anesthetics. Data points illustrate *I*/*I*_0_ as functions of each local anesthetic concentration. Grey circles represent lidocaine; red circles represent prilocaine; and blue circles represent articaine. Curves (solid lines) were fitted according to the equation described in the text, except for the articaine. Each data point represents the mean ± SD of data from six separate experiments. Significant differences between data points are indicated using asterisks. *p<0.05. D.W., distilled water; IC50, the 50% inhibitory concentration; ESR, electron spin resonance; SD, standard deviation

To investigate the antioxidant properties of dental local anesthetics, we measured the O_2_^•−^ -scavenging activities of lidocaine, prilocaine, and articaine. Lidocaine and prilocaine significantly inhibited O_2_^•−^ generation from distilled water in a concentration-dependent manner (Figure 3A, 3B), with IC_50_ values of 0.033 and 0.057 wt%, respectively (Figure 3D). However, articaine did not significantly inhibit/scavenge O_2_^•−^ (Figure 3C, 3D).

## Discussion

We investigated the HO^•^- and O_2_^•−^-scavenging activities of three dental local anesthetics (lidocaine, prilocaine, and articaine). Lidocaine and prilocaine effectively scavenged HO^•^ and O_2_^•−^ in a concentration-dependent manner. In contrast, articaine scavenged only HO^•^ in a concentration-dependent manner but did not scavenge O_2_^•−^ at the tested concentrations.

Although several studies have shown the antioxidant effects of local anesthetics, reports on the direct capture of ROS are limited. For example, studies using the ESR spin-trapping technique showed that lidocaine scavenged HO^•^ in a concentration-dependent manner, with IC_50_ of approximately 80 µM [26,27]. Considering that the molecular weight of lidocaine is 288.81, its IC_50_ (80 µM) corresponded to 0.0023%. Overall, the results of the present study are consistent with the previous reports (Figure 2A, 2D) [26,27]. However, the concentrations at which the antioxidant effect against HO^•^ was detected were lower than those typically used in clinical practice (0.5-2%). In dental practice, 2% lidocaine containing epinephrine (1:80,000) prepared in dedicated cartridges is typically used. Notably, the concentration of lidocaine in the oral mucosa was diluted to approximately 360-120 μg/g after 10-60 min of injection with 0.5 mL of the local anesthetic [28]. Considering that the IC_50_ of lidocaine against HO^•^ in the present study was approximately 0.029% (Figure 2D), may sufficiently scavenge HO^•^ even when injected locally. In contrast, lidocaine did not reportedly scavenge O_2_^•−^ [26,27]. Additionally, a comparative study of eight different local anesthetics showed that high concentrations of local anesthetics are necessary to effectively scavenge O_2_^•−^ in human neutrophils [29]. These previous findings suggest that lidocaine has a low O_2_^•−^ scavenging potential. Moreover, our study showed that lidocaine had a maximum O_2_^•−^ scavenging rate of approximately 40% (Figure 3D), thereby confirming its limited scavenging activity.

Notably, we demonstrated the direct ROS scavenging activities of prilocaine and articaine. A study on lipid peroxidation using a liposome membrane system showed that prilocaine has considerably lower antioxidant activity than other local anesthetics [30]. Additionally, Hattori M et al. showed that O_2_^•−^ inhibitory effect of prilocaine in neutrophils was as low as that of lidocaine [29]. Moreover, a study using the xanthine-xanthine oxidase-induced chemiluminescence showed that prilocaine inhibited O_2_^•−^ levels in a concentration-dependent manner but did not inhibit HO^•^ [31]. In this study, similar to lidocaine, prilocaine showed high HO^•^- and O_2_^•−^-scavenging activity (Figures 2, 3). Additionally, articaine effectively scavenged HO^•^ in a concentration-dependent manner (Figure 2C, 2D), but did not scavenge O_2_^•−^ (Figure 3C, 3D). Articaine differs from other amide-based local anesthetics as it contains an ester bond and a thiophene ring [32]. Therefore, articaine has higher lipid solubility compared with other local dental anesthetics and undergoes hydrolysis by nonspecific cholinesterase in the body [32]. In a study using the xanthine-xanthine oxidase-induced chemiluminescence, high articaine concentrations showed reactivity against O_2_^•−^ [31]. Although whether articaine can effectively scavenge O_2_^•−^ is debatable, the differences in chemical structure may stiff affect its O_2_^•−^-scavenging ability.

Local anesthetics are used to manage local pain in clinical settings and dental practice. Lidocaine eliminates ROS through direct scavenging, ROS-generating enzyme inhibition, mitochondrial protection, inflammatory pathway modulation, and antioxidant defense upregulation [14-18, 33-35]. Therefore, these multiple mechanisms may have contributed to the antioxidant properties of prilocaine and articaine in the present study, and the proactive use of dental local anesthetics may potentially mitigate oxidative injury and inflammatory damage. Recently, a study using lipid raft model membranes reported that local anesthetic-induced lipid raft disruption may indirectly affect the activity of raft-associated proteins, leading to anesthetic action [36]. Lipid peroxidation, which leads to ROS production, may be one of the mechanisms underlying lipid raft disruption.

Despite the promising findings, this study had several limitations. First, we only focused on the chemical reaction of ROS-scavenging activities of local anesthetics. Second, although the antioxidant mechanism of lidocaine has been explored, the mechanisms of action of prilocaine and articaine remain speculative. Third, we did not investigate the potential clinical implications of the observed ROS-scavenging activities, and further research is needed to determine whether these properties translate into beneficial clinical outcomes. Finally, considering that this study is in vitro, the results may not be directly applicable to the complex environment of the human body [22]. Despite these limitations, the present study provides valuable insights into the potential antioxidant properties of lidocaine, prilocaine, and articaine. Further research is needed to fully understand the mechanisms and clinical implications of these findings.

## Conclusions

We investigated the ROS-scavenging activities of lidocaine, prilocaine, and articaine, focusing on their effects on HO^•^ and O_2_^•−^. These activities were concentration-dependent, except for articaine's O_2_^•−^-scavenging effect. Notably, antioxidant effects were observed at sub-clinical concentrations. While currently used solely for pain relief, our findings suggest these dental local anesthetics may have potential applications in treating oxidative stress-associated oral diseases, expanding their utility beyond pain management.
